# Fabrication and Photocatalytic Property of Novel SrTiO_3_/Bi_5_O_7_I Nanocomposites

**DOI:** 10.1186/s11671-018-2558-6

**Published:** 2018-05-11

**Authors:** Yongmei Xia, Zuming He, Jiangbin Su, Ya Liu, Bin Tang

**Affiliations:** 1 Jiangsu Key Laboratory of Advanced Material Design and Additive Manufacturing, School of Materials and Engineering, Jiangsu University of Technology, Changzhou, 213001 China; 2 Huaide College, Changzhou University, Jingjiang, 214500 China; 3 School of Mathematics&Physics, Changzhou University, Changzhou, 213164 China; 4 Jiangsu Key Laboratory of Materials Surface Science and Technology, Changzhou University, Changzhou, 213164 China

**Keywords:** SrTiO_3_, Bi_5_O_7_I, Nanocomposite, Photocatalytic, Mechanism

## Abstract

The novel SrTiO_3_/Bi_5_O_7_I nanocomposites were successfully fabricated by a thermal decomposition approach. The as-prepared samples were characterized by XRD, XPS, SEM, EDS, FTIR, DRS and PL spectra. The results show that the SrTiO_3_/Bi_5_O_7_I nanocomposites are composed of perovskite SrTiO_3_ nanoparticles and tetragonal Bi_5_O_7_I nanorods. The SrTiO_3_/Bi_5_O_7_I nanocomposites exhibit an excellent photocatalytic performance for the degradation of RhB solution under simulated solar light irradiation, which is superior to that of pristine Bi_5_O_7_I and SrTiO_3_. In particular, the 30 wt% SrTiO_3_/Bi_5_O_7_I nanocomposite is found as the optimal composites, over which the dye degradation reaches 89.6% for 150 min of photocatalysis. The photocatalytic degradation rate of the 30 wt% SrTiO_3_/Bi_5_O_7_I nanocomposite is found to be 3.97 times and 12.5 times higher than that of bare Bi_5_O_7_I and SrTiO_3_, respectively. The reactive species trapping experiments suggest that $\bullet {\mathrm{O}}_2^{-}$ and holes are the main active species responsible for the RhB degradation. In addition, the PL spectra elucidate the effective separation of photoinduced electron-hole pairs. Further, the possible photocatalytic mechanism of the SrTiO_3_/Bi_5_O_7_I nanocomposites is also elucidated based on the experimental evidences.

## Background

Dyes from textile or dyestuff industries have aroused much concern for the impact on the quality of water resources and the toxic and carcinogenic degradation products [1]. Therefore, more competent treatment techniques are needed for the complete elimination of dyes form wastewater. Several conventional methods involving physical, chemical, and biological methods have been applied for dye remediation from wastewater [2]. These methods can remove dyes from wastewater, but they are often expensive, inefficient, and produce secondary waste products [3, 4]. Among various dye wastewater treatment technologies, semiconductor-based photocatalysis has received a great interest and attracted worldwide attention [5–7]. This is because it utilizes solar energy for the decomposition of dye pollutants, whose source of energy is abundant, inexhaustible, non-polluting, and free [8, 9]. At present, TiO_2_ is the most widely used semiconductor photocatalyst due to its high photoactivity, low cost, chemical and photochemical stability, non-toxicity, and environmentally friendly features. However, it showed a very low photocatalytic activity under visible light irradiation due to its wide band gap of 3.2 eV and the rapid recombination of photogenerated carriers [10]. To effectively solve the above mentioned problems, much work has been devoted to the surface modification or the combination of semiconductor photocatalysts [11]. Nevertheless, the development of novel and highly efficient photocatalysts still remains a major challenge [12].

Bi_5_O_7_I is a newly found p-type semiconductor, which shows a relatively more positive valence band (VB) level than other bismuth oxyiodides by providing more photo-excited holes and subsequently facilitates the separation of photogenerated carriers [13]. Therefore, the Bi_5_O_7_I photocatalyst exhibits a high activity for the photodegradation of Rhodamine B (RhB) in water and acetaldehyde under visible light irradiation [14]. Unfortunately, the practical application of Bi_5_O_7_I photocatalyst in the environmental decontamination is still limited, which is attributed to its low transfer efficiency caused by the recombination of photogenerated electrons and holes [15]. For the purpose of further improving the photoactivity of Bi_5_O_7_I, many attempts have been carried out such as doping with metals or non-metals [16], or coupling with other semiconductors. For instance, Huang et al. synthesized g-C_3_N_4_/Bi_5_O_7_I heterojunction via a co-crystallization method, and the composite exhibited a degradation rate 2.9 times higher than that of pure Bi_5_O_7_I [17]; Cheng et al. fabricated Bi_5_O_7_I/Bi_2_O_3_ composite via chemical etching method, which showed a high photocatalytic activity in decomposition of malachite green [18]; Hu et al. reported that a composite comprising n-type Sr_2_TiO_4_ and p-type Bi_5_O_7_I showed an enhanced photoactivity because of the inhibition of electron-hole recombination [19]; Cui et al. fabricated AgI/Bi_5_O_7_I hybrid via a simple one-step ionic reaction, and the AgI/Bi_5_O_7_I composite enhanced the photocatalytic activity [20], and so on. These results showed that Bi_5_O_7_I-based composites exhibited an enhanced photocatalytic performance under visible light irradiation. Therefore, we can fabricate Bi_5_O_7_I-based composites through coupling to another semiconductor with suitable conduction band (CB) and VB positions as a promising visible-light-driven photocatalyst. Among various candidates, strontium titanate (SrTiO_3_) is an n-type semiconductor material, which has been extensively studied because of its many excellent properties, e.g., thermal stability, good heat resistance, corrosion, and resistance [21–23]. Pure SrTiO_3_ only absorbs UV light due to its wide band gap of 3.1~3.4 eV [24]. Fortunately, the VB of SrTiO_3_ is positioned between the CB and VB of Bi_5_O_7_I, while its CB is positioned above the CB of Bi_5_O_7_I. Considering the structural merits of Bi_5_O_7_I, combination of SrTiO_3_ with Bi_5_O_7_I to form the SrTiO_3_/Bi_5_O_7_I composite may be a viable and advisable way to realize the high photocatalytic activity.

In this work, a series of SrTiO_3_/Bi_5_O_7_I nanocomposite photocatalysts were first synthesized. Their crystal phase, microstructure, and optical properties were investigated by a series of techniques. The SrTiO_3_/Bi_5_O_7_I nanocomposites displayed an enhanced photocatalytic performance in the degradation of Rhodamine B (RhB) solution under simulated solar light irradiation. Further, the possible photocatalytic mechanism of the SrTiO_3_/Bi_5_O_7_I nanocomposites was also elucidated based on the experimental evidences.

## Methods

### Preparation of SrTiO_3_/Bi_5_O_7_I Composites

SrTiO_3_ nanoparticles and SrTiO_3_/BiOI composites were firstly synthesized via a sol-gel method as described in the literature [25, 26]. SrTiO_3_/Bi_5_O_7_I composites were then synthesized by a thermal decomposition route. All the chemical reagents were used directly for the experiments without any further purification. During the thermal decomposition, the as-prepared SrTiO_3_/BiOI composites were placed into a tube furnace, and the heating program was set as follows: ramping at 5 °C min^− 1^ to 500 °C continuously and holding at 500 °C for 3 h. Then, the furnace was naturally cooled to room temperature to obtain 10 wt% SrTiO_3_/Bi_5_O_7_I nanocomposite. Other SrTiO_3_/Bi_5_O_7_I nanocomposite materials with different SrTiO_3_ content were fabricated by the similar method.

### Sample Characterization

The crystal structures of the synthesized samples were characterized using X-ray diffraction (XRD) with Cu K_α_ radiation (D/max-2500, Rigaku). The morphology of the samples was investigated by an ultrahigh resolution field-emission scanning electron microscope (FE-SEM; SUAPR55, Germany Zeiss) with energy-disperse X-ray spectroscopy (EDS). The surface elemental component and the chemical state of the samples were analyzed by X-ray photoelectron spectroscopy (XPS; Axis Ultra DLD, Kratos Analytical, UK) with a monochromatized Al K_α_ X-ray source (hν = 1486.6 eV). The ultraviolet-visible (UV-vis) diffuse reflectance spectra (DRS) were obtained using a UV-vis spectrophotometer (UV-2450, Shimadzu). The functional groups on the surface of the samples were investigated in a Nicolet iS50 fourier-transform infrared spectroscopy (FTIR; Thermo Fisher Scientific, USA). The photoluminescence (PL) emission spectra were measured on a LH110911 steady-state fluorescence spectrometer.

### Photocatalytic Evaluation Studies

The photocatalytic activity of the materials was evaluated via the decomposition of RhB under simulated solar light (UV light) irradiation in a photoreaction apparatus. After 30 min adsorption in darkness, the adsorption-desorption is at an equilibrium between photocatalyst and RhB molecules. A 500 W xenon lamp was used as a simulated solar light (UV lamp) source. One hundred milligrams of photocatalyst was completely dispersed in 100 mL RhB solution (20 mg/L). During each photocatalytic experiment, 3 mL of the suspension was pipet out every 30 min and centrifuged to remove catalyst particles. The concentration of the RhB was measured using UV-vis spectrophotometer.

## Results and Discussion

### XRD Analysis

**Fig. 1:**
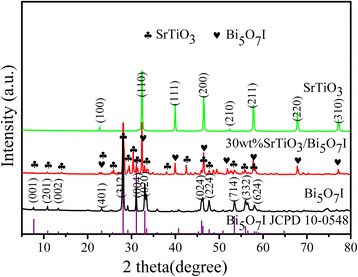
XRD patterns of pure Bi_5_O_7_I, SrTiO_3_ and 30 wt% SrTiO_3_/Bi_5_O_7_I nanocomposite

The powder XRD patterns provide the crystal structure and phase information of the synthesized samples, as shown in Fig. 1. The SrTiO_3_ sample is highly crystallized with a perovskite structure (JCPDS no. 35-0734). The diffraction peaks at the 2*θ* values of 22.75°, 32.39°, 39.95°, 46.47°, 52.34°, 57.78°, 67.82°, and 77.18° can be indexed to (100), (110), (111), (200), (210), (211), (220), and (310) crystal planes, respectively [27]. No other specific diffraction peak is detected. From the XRD pattern of pure Bi_5_O_7_I, it can be seen that the main diffraction peaks at 7.71°, 13.31°, 15.38°, 23.19°, 28.08°, 31.09°, 33.43°, 46.28°, 47.69°, 53.45°, 56.51°, and 58.02° are conforming to the (001), (201), (002), (401), (312), (004), (020), (024), (224), (714), (332), and (624) planes of the Bi_5_O_7_I (JCPDS No. 10-0548), respectively [28]. The strongest peak is corresponding to the (312) crystal plane of Bi_5_O_7_I. From the XRD pattern of the 30 wt% SrTiO_3_/Bi_5_O_7_I nanocomposite, it can be found that all prominent diffraction peaks are arising from tetragonal Bi_5_O_7_I and perovskite SrTiO_3_. There are no other obvious peaks of impurity observed, which indicates that the Bi_5_O_7_I and SrTiO_3_ phases coexist in the composite.

### XPS Analysis

**Fig. 2:**
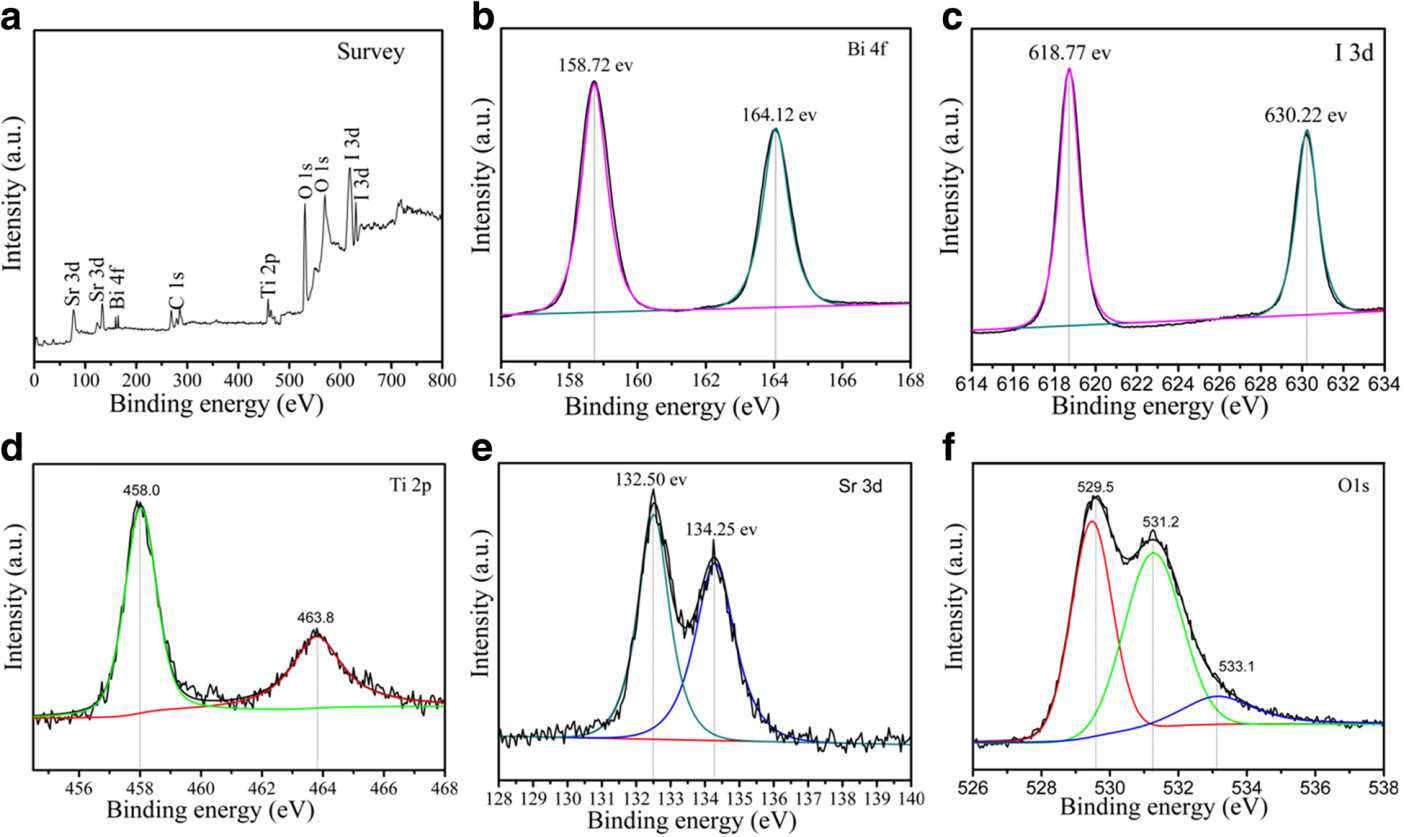
XPS patterns of the 30 wt% SrTiO_3_/Bi_5_O_7_I nanocomposites: **a** survey, **b** Bi 4f, **c** I 3d , **d** Ti 2p, **e** Sr 3d, and **f** O1s

The XPS measurements provide further information for the evaluation of the surface elemental composition and purity of the 30 wt% SrTiO_3_/Bi_5_O_7_I nanocomposite. The binding energy obtained in the XPS analysis was corrected for specimen charging by referencing C 1 s to 284.65 eV, and the results are displayed in Fig. 2. The XPS survey scan spectrum of the composite is shown in Fig. 2a, which reveals the existence of Ti, Sr, Bi, I, and O elements in the composite. The two strong peaks at 159.02 and 164.25 eV are respectively assigned to Bi 4f_5/2_ and Bi 4f_7/2_ peaks of Bi^3+^ in the SrTiO_3_/Bi_5_O_7_I nanocomposites as shown in Fig. 2b [29]. In the XPS spectra of I 3d shown in Fig. 2c, the two strong peaks at 617.88 and 630.22 eV, corresponding to I 3d_5/2_ and I 3d_3/2_, respectively, suggest the − 1 oxidation state of iodine [30]. As shown in Fig. 2d, the binding energies of Ti 2p_3/2_ and Ti 2p_1/2_ correspond to the peaks at 457.90 and 463.80 eV in the spectrum of Ti 2p, respectively. The peak separation between the Ti 2p_3/2_ and Ti 2p_1/2_ is 5.90 eV, which indicates a + 4 oxidation state of Ti in the SrTiO3/Bi_5_O_7_I composites [31]. In Fig. 2e, the peaks at 132.50 and 134.25 eV correspond to the binding energies of Sr 3d_5/2_ and Sr 3d_3/2_, respectively, indicating its existence in the Sr^2+^ state [32]. In Fig. 2f, the peaks at 529.65 and 531.25 eV are attributed to O 1 s. The peak at 529.65 eV is ascribed to the lattice oxygen of SrTiO_3_/Bi_5_O_7_I nanocomposites, and the peak at 531.25 eV is generally attributed to the chemisorbed oxygen caused by oxygen vacancies [33]. The XPS result further confirms the formation of SrTiO_3_/Bi_5_O_7_I nanocomposites, and intimate integration has been achieved, which agrees well with the XRD results.

### SEM and EDS Analysis

**Fig. 3:**
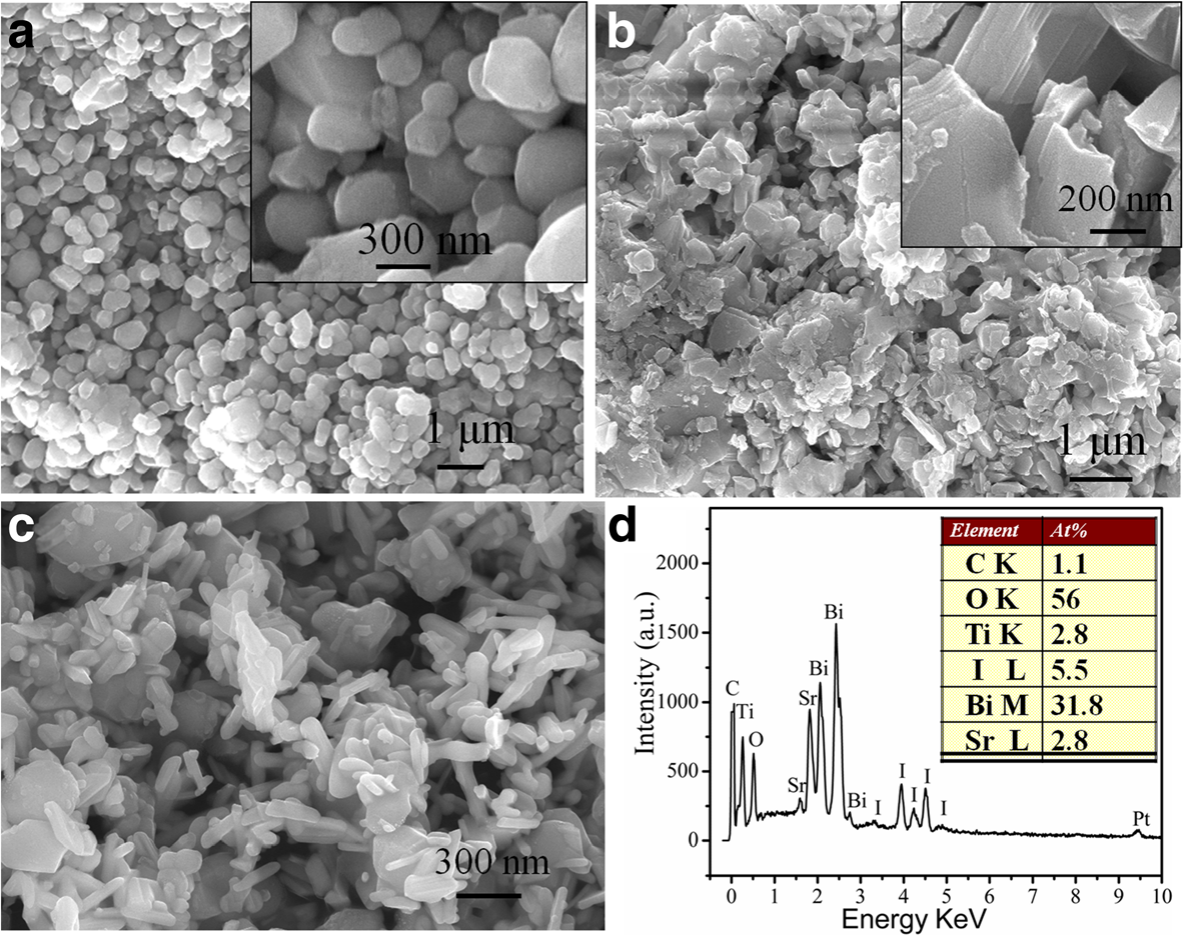
SEM images of **a** pure SrTiO_3_, **b** pure Bi_5_O_7_I, **c** 30 wt% SrTiO_3_/Bi_5_O_7_I nanocomposite, and **d** EDS spectrum of 30 wt% SrTiO_3_/Bi_5_O_7_I nanocomposite

The surface compositions and morphologies of as-prepared pure SrTiO_3_, Bi_5_O_7_I and 30 wt% SrTiO_3_/Bi_5_O_7_I nanocomposite were observed by FE-SEM. As seen in Fig. 3a, pure SrTiO_3_ is composed of spheroidal or spherical particles with diameters in the range of 50~300 nm. The smaller sized SrTiO_3_ particles are obviously aggregated together to some extent. In Fig. 3b, for the Bi_5_O_7_I nanosheets, they have an average size about 1 μm and a thickness in the range of 80~100 nm, which is similar to that reported previously [13]. In contrast, after the combination the Bi_5_O_7_I is not of nanosheets but of nanorods morphology, which is constructed by plenty of nanorods, as shown in Fig. 3c. For the Bi_5_O_7_I nanorods, the length is in the range of 100~300 nm and the average diameter is about 80 nm. It can be clearly seen that SrTiO_3_ particles are tightly adhered on the surface of Bi_5_O_7_I nanorods, and it is thought to be favorable for the photocatalytic performance. Furthermore, EDS was further used to analyze the chemical composition of the 30 wt% SrTiO_3_/Bi_5_O_7_I nanocomposite. As shown in Fig. 3d, the observed C signal can be derived from the conductive adhesive which is used to fix the sample. It is noted that EDS is suitably used for the quantitative determination of the content of heavy elements (e.g., Bi, Ti, I, and Sr), but not for the light elements (e.g., P and O) [34]. The atomic ratio of Bi to I is obtained as 11/63 from the EDS spectrum, which agrees well with the Bi/I atomic ratio of Bi_5_O_7_I phase. The atomic ratio of Sr/Bi is very close to 1/12.5, implying that SrTiO_3_ phase accounts for about 30% of the total molar content of the composite.

### Optical Properties Analysis

**Fig. 4:**
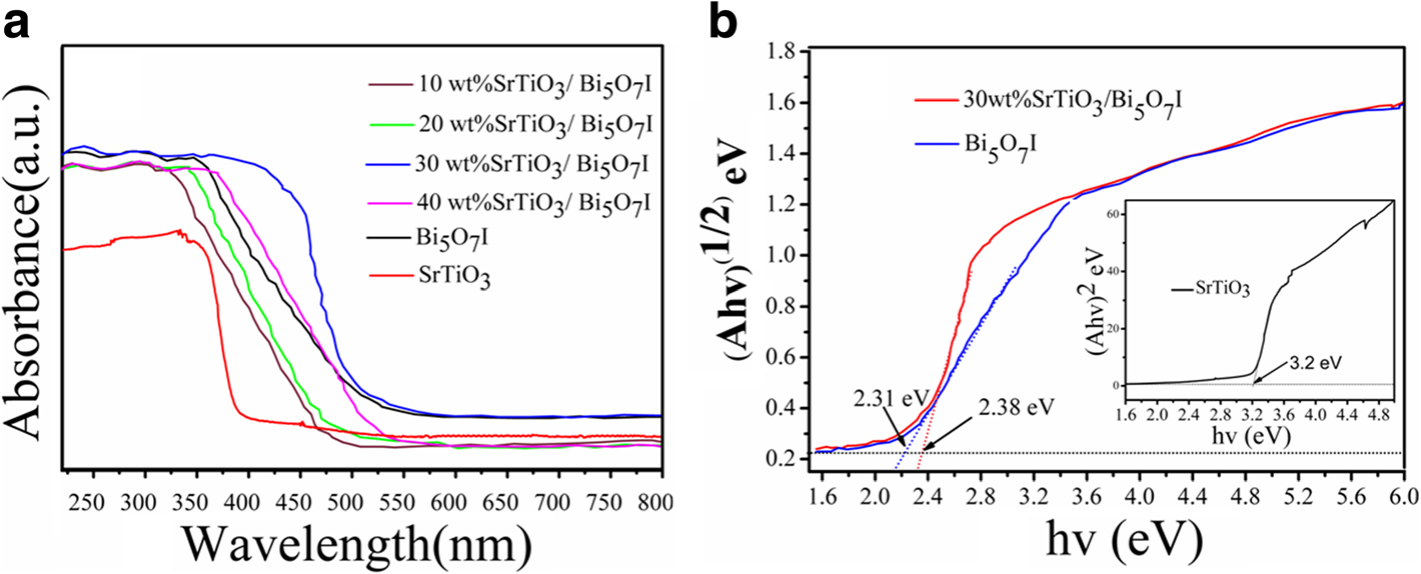
**a** DRS spectra of SrTiO_3_/Bi_5_O_7_I nanocomposites and **b** the Tauc plot of (Ahν)^1/2^ versus hν the pure Bi_5_O_7_I and 30 wt% SrTiO_3_/Bi_5_O_7_I nanocomposite, the Tauc plot of (Ahν)^2^ versus hν the pure SrTiO_3_ inset picture of **b**

The UV-vis DRS spectra of the different catalysts are shown in Fig. 4a. The pure SrTiO_3_ exhibits an absorption band edge at 380 nm in the UV region, which might be attributed to the wide band gap energy [35, 36]. The Bi_5_O_7_I shows a much longer absorption edge of 520 nm, which can respond to the visible light. The absorption edge of the SrTiO_3_/Bi_5_O_7_I nanocomposites are 480~520 nm. Compared with pure SrTiO_3_, after coupling with Bi_5_O_7_I nanosheets, the absorption peak intensity of the SrTiO_3_/Bi_5_O_7_I nanocomposites enhanced significantly.

Based on the absorption spectra, the *E*_g_ of the semiconductor can be calculated from the Aһν = A (*һν* − *E*_g_)^*n*/2^ equation [37]. The values of *n* for SrTiO_3_ and Bi_5_O_7_I are 4 and 1, respectively. The band gap energy of the SrTiO_3_ can be estimated from the plot (Aһν)^2^ versus ploton energy (һν), and the band gap energy of the Bi_5_O_7_I can be estimated from the plot (Aһν)^1/2^ versus һν. The intercept of the tangent to the *X* axis gives an approximation of the band gap energy of the samples as displayed in Fig. 4b. The values of band gap energy of pure Bi_5_O_7_I, 30 wt% SrTiO_3_/Bi_5_O_7_I nanocomposite and pure SrTiO_3_ are about 2.31, 2.38, and 3.2 eV, respectively, which are consistent with the reported values in the relevant literature [38, 39].

### FTIR Spectroscopy Analysis

**Fig. 5:**
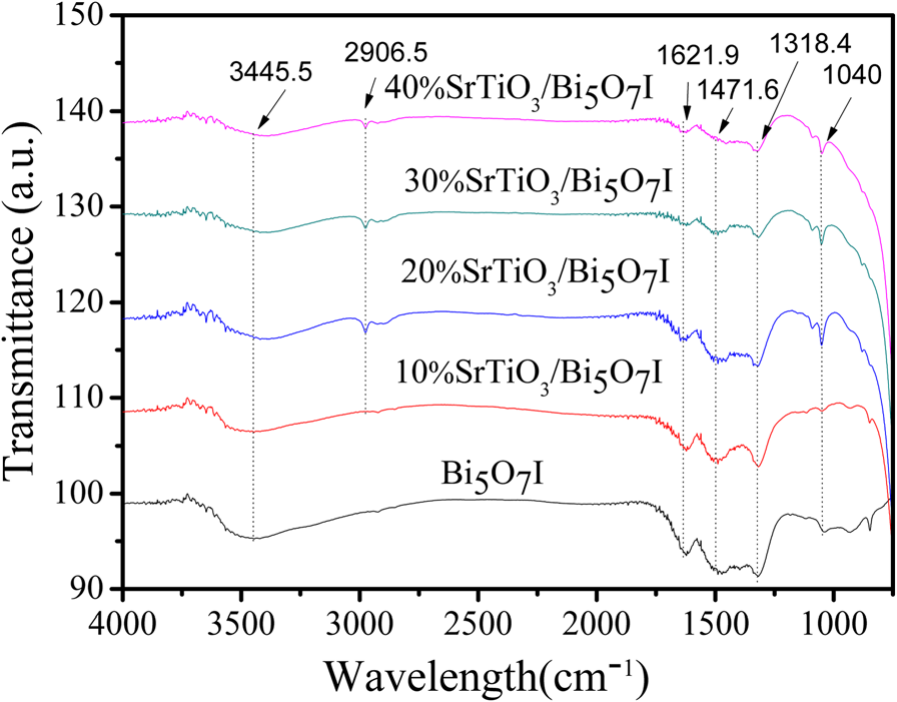
FTIR spectra of the pure Bi_5_O_7_I and SrTiO_3_/Bi_5_O_7_I nanocomposites

The Bi_5_O_7_I and SrTiO_3_/Bi_5_O_7_I nanocomposites were further characterized using FTIR spectroscopy to analyze their chemical bonding. As shown in Fig. 5. It can be seen that in almost all samples the adsorption bands of 3445.5 and 1621.9 cm^−1^were due to the O–H stretching vibration and deformation vibration of chemisorbed water molecules [40]. The band at 2906.5 cm^−1^ is ascribed to the Ti–O stretching vibration [41]. The other peaks in the range of 1471.6–500 cm^−1^ correspond to the stretching and deformation modes involving in Bi–O bonds [42].

### Photocatalytic Activity

**Fig. 6:**
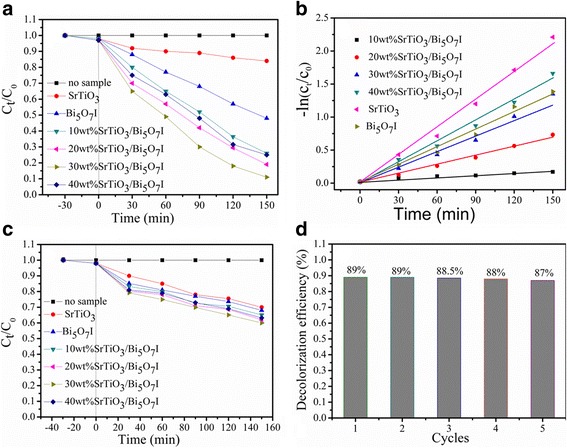
**a** Photocatalytic degradation of RhB solution for all samples under simulated solar light irradiation. **b** Plots of -ln(C_t_/C_0_) vs time for all samples. **c** Photocatalytic degradation of RhB solution for all samples under UV light illumination. **d** Cycling degradation efficiency of RhB over the SrTiO_3_/Bi_5_O_7_I nanocomposites

As Bi_5_O_7_I and SrTiO_3_ have very distinct photo absorption and SrTiO_3_ mainly responds to UV light, simulated solar light and UV light under the same condition are separately employed as the light source to explore the photocatalytic performance of Bi_5_O_7_I, SrTiO_3_, and SrTiO_3_/Bi_5_O_7_I nanocomposites. Figure 6a displays the degradation curves of RhB under simulated solar light irradiation. It is observed that RhB is stable and hardly decomposed without the catalyst under simulated solar irradiation for 150 min. The pure SrTiO_3_ shows moderate catalytic activity, and only 18% RhB reduction was achieved after 150 min irradiation. It is attributed to the low light absorption of SrTiO_3_ in the visible light region or its large band gap energies. Pure Bi_5_O_7_I displays a very distinct activity which degrades over 52% of RhB in 150 min. Compared with pure SrTiO_3_ and Bi_5_O_7_I, the SrTiO_3_/Bi_5_O_7_I nanocomposites display a significantly enhanced photocatalytic activity under the same condition. With the increase of SrTiO_3_ content from 10 to 40%, the photocatalytic activity of the composites increases firstly and then decreases, and the highest photocatalytic activity is observed for the 30 wt% SrTiO_3_/Bi_5_O_7_I nanocomposite. For this optimal composite, the dye degradation reaches approximately 89.6% under simulated solar light irradiation for 150 min. Such a high activity can be ascribed to the photogenerated electrons having a faster mobility and separation.

To further understand the reaction kinetics of RhB photocatalytic degradation for different photocatalysts, the photocatalytic degradation efficiency were computed using the following equation: ln(*C*_0_/*C*_*t*_) = *K*_*app*_*t*, where *C*_0_, *C*_t_, and *K*_app_ are representative of the initial concentration, the concentration at time t, and the apparent pseudo-first-order rate constant, respectively [43]. The -ln(C_t_/C_0_) exhibits a well linear relationship with irradiation time and the photocatalytic reaction belongs to the pseudo-first-order reaction as shows in Fig. 6b. The *k*_app_ values obtained for Bi_5_O_7_I, SrTiO_3_, 10 wt% SrTiO_3_/Bi_5_O_7_I, 20 wt% SrTiO_3_/Bi_5_O_7_I, 30 wt% SrTiO_3_/Bi_5_O_7_I, and 40 wt% SrTiO_3_/Bi_5_O_7_I nanocomposites are 1.16 × 10^−3^, 4.88 × 10^−3^, 9 × 10^−3^, 1.06 × 10^−2^, 1.45 × 10^−2^, and 9.24 × 10^−3^ min^−1^, respectively. It is conspicuous that the 30 wt% SrTiO_3_/Bi_5_O_7_I nanocomposite manifests the maximum photocatalytic reaction rate constant, which is about 2.97 times higher than that of bare Bi_5_O_7_I, and 12.5 times higher than that of pure SrTiO_3_.

The RhB degradation curves over all samples under UV light irradiation are further shown in Fig. 6c. It can be clearly seen that the phenomenon is similar to that in Fig. 6a. However, all samples present a very low photocatalytic efficiency due to the little absorption of UV light, and dye sensitization has an effect on the photocatalytic activity. As shown in Fig. 6a, the 30 wt% SrTiO_3_/Bi_5_O_7_I nanocomposite still displays the best activity; however, the photodegradation of RhB is only 40% within 150 min. These results demonstrate that the SrTiO_3_/Bi_5_O_7_I nanocomposites possess more efficient photocatalytic activity under simulated solar light irradiation.

The stability and reusability of the 30 wt% SrTiO_3_/Bi_5_O_7_I nanocomposite was conducted by repeating the tests for the RhB degradation. After each cycle, the SrTiO_3_/Bi_5_O_7_I nanocomposites were reused in the next cycle before being collected by centrifugation, washed several times with deionized water and ethyl alcohol, and finally dried at 80 °C for 3 h. As shown in Fig. 6d, the photocatalytic activity of the composite does not decrease obviously even after the fifth recycle under simulated solar irradiation, which suggests a good stability for recycling the 30 wt% SrTiO_3_/Bi_5_O_7_I nanocomposite.

### Photocatalytic Mechanism Discussion

**Fig. 7:**
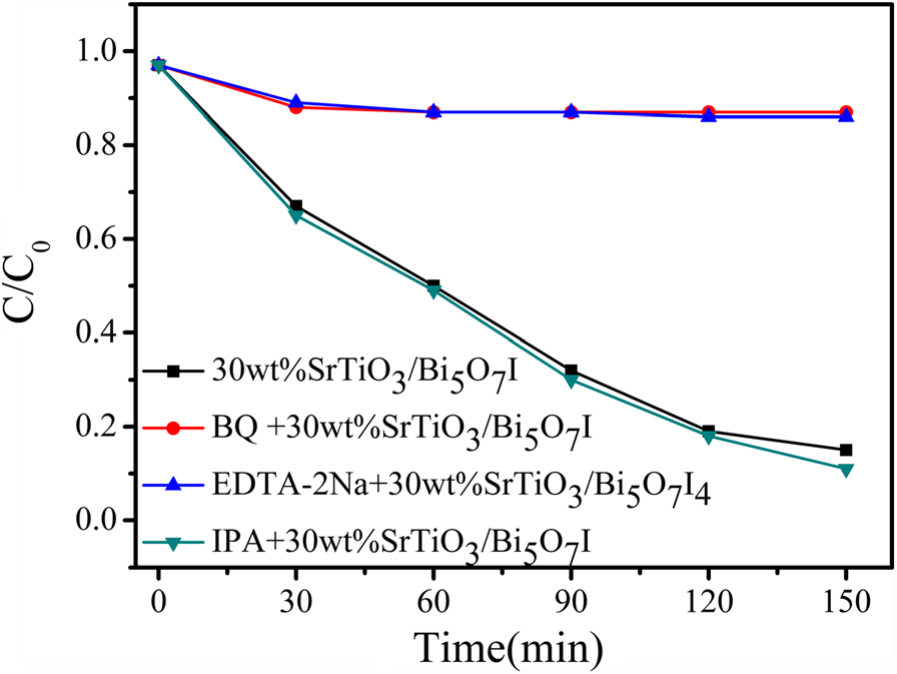
The degradation efficiency constant of RhB over the 30 wt% SrTiO_3_/Bi_5_O_7_I nanocomposite in the presence of various scavengers

In order to gain some insight into the active species involved in the photodegradation of SrTiO_3_/Bi_5_O_7_I nanocomposites, we carried out reactive species trapping experiments over the 30 wt% SrTiO_3_/Bi_5_O_7_I nanocomposite to ascertain the main active species in photocatalytic reaction. As shown in Fig. 7, the addition of the isopropanol (IPA) has almost no effect on the RhB degradation over the SrTiO_3_/Bi_5_O_7_I nanocomposites, which indicates that no •OH radicals are generated. On the contrary, a significant decrease in the dye degradation is observed after the addition of benzoquinone (BQ) or ethylenediaminetetraacetic acid disodium salt (EDTA-2Na), which implies that $\bullet {\mathrm{O}}_2^{-}$ and holes are the primary reactive substances for RhB photodegradation.

**Fig. 8:**
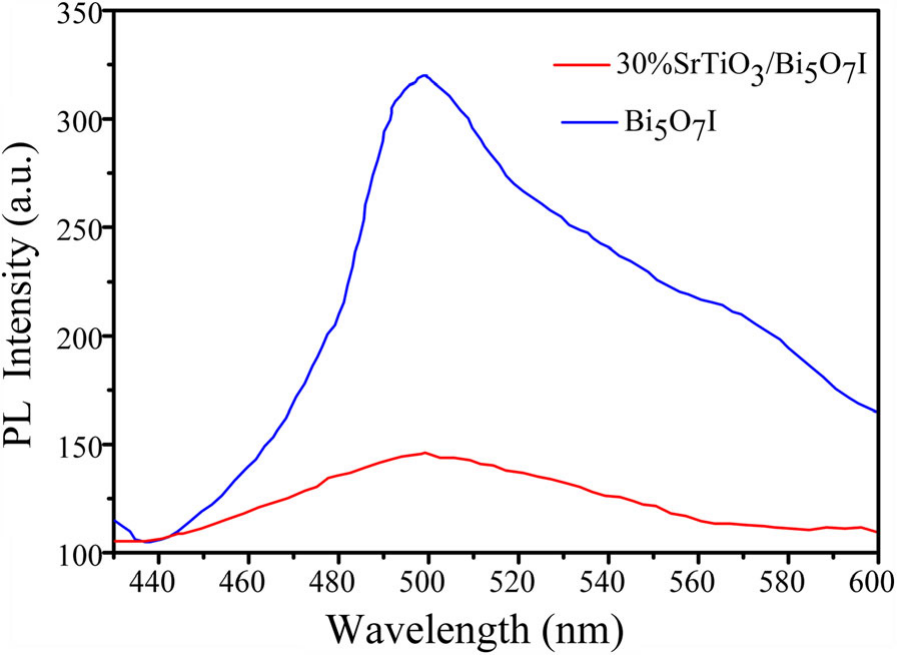
PL spectra of pure Bi_5_O_7_I nanosheets and 30 wt% SrTiO_3_/Bi_5_O_7_I nanocomposite

In order to investigate the charge separation of the as-prepared photocatalysts, the PL spectroscopy was further introduced. Figure 8 shows the comparison of the PL spectra between Bi_5_O_7_I and 30 wt% SrTiO_3_/Bi_5_O_7_I nanocomposite under the excitation at 320 nm. The PL spectra of both samples are characterized by a peak at 497 nm, which is attributed to the emission of band gap transition energy of Bi_5_O_7_I. However, there is a sharp decrease in the intensity of the 30 wt% SrTiO_3_/Bi_5_O_7_I nanocomposite. This phenomenon demonstrates an efficient separation of the photogenerated carriers within the composite between SrTiO_3_ and Bi_5_O_7_I.

To better understand the mechanism of the enhanced photocatalytic activity of the SrTiO_3_/Bi_5_O_7_I nanocomposites under simulated solar light irradiation, the corresponding CB and VB positions for SrTiO_3_ and Bi_5_O_7_I are theoretically computed according to ${E}_{VB}=\chi -{E}_0+\frac{1}{2}{E}_g$ and *E*_*CB*_ = *E*_*VB*_ − *E*_*g*_, where *E*_VB_ is the VB potential, *E*_CB_ is the CB potential, *E*_0_ is the energy of free electrons on the hydrogen scale (ca. 4.5 eV), *E*_g_ is the band gap energy, and χ is calculated as the geometric mean of the Mulliken electronegativities of the components in the semiconductor [44]. Therefore, the *E*_VB_ and *E*_CB_ of Bi_5_O_7_I were computed to be 2.92 and 0.56 eV, whereas the energies of SrTiO_3_ were about 2.03 and − 1.15 eV, respectively. These two semiconductors have suitable band potentials and thus can construct a composite structure.

**Fig. 9:**
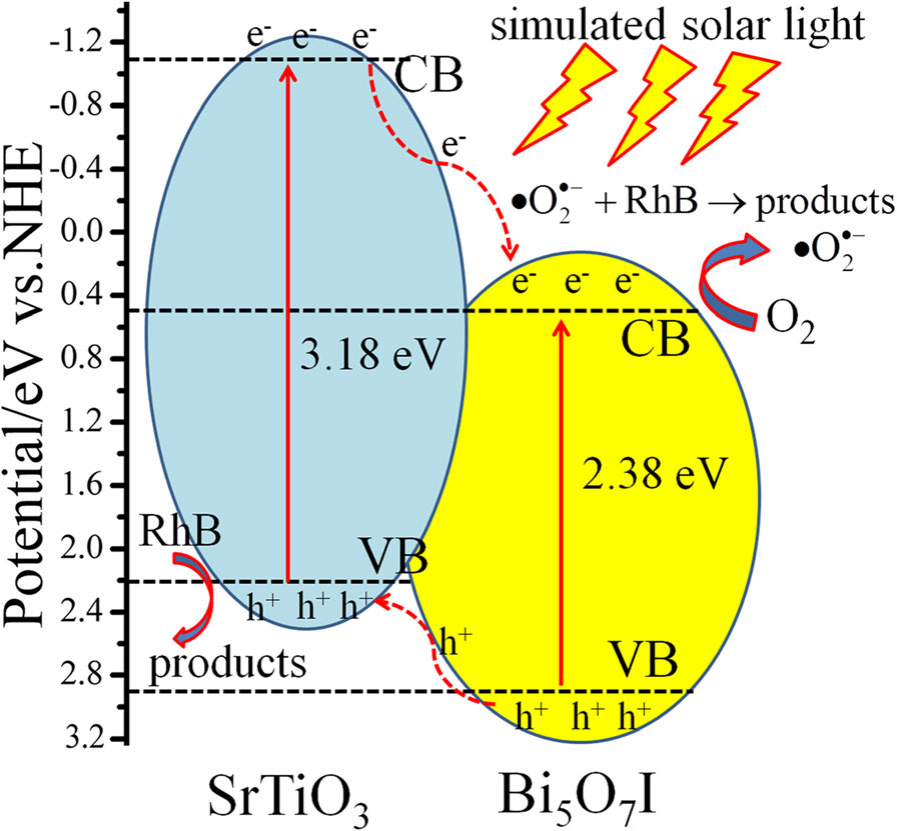
The schematic energy band diagram and possible photocatalytic process of SrTiO_3_/Bi_5_O_7_I nanocomposites

According to the above results, a schematic illustration of energy bands matching between SrTiO_3_ and Bi_5_O_7_I and possible ways of charges transfer are depicted in Fig. 9. Both SrTiO_3_ and Bi_5_O_7_I are excited under simulated solar light (UV light), and the electrons in the VB of both SrTiO_3_ and Bi_5_O_7_I would be excited to the CB, the holes remained in its VB. As the CB potential of SrTiO_3_ (− 1.15 eV) is more negative than that of Bi_5_O_7_I (+ 0.56 eV), the electrons of SrTiO_3_ are easily injected into the CB of Bi_5_O_7_I. The photogenerated electrons could react with O_2_ to produce active oxygen species superoxide radical ($\bullet {\mathrm{O}}_2^{-}$), which then induces the RhB degradation [45]. On the other hand, the holes in the VB of Bi_5_O_7_I migrate to the VB of SrTiO_3_, resulting in an effective separation of the photoinduced electrons and holes. In this way, the photogenerated electrons and holes are separated effectively in the SrTiO_3_/Bi_5_O_7_I nanocomposites. The VB of SrTiO_3_ is 2.23 eV, lower than the redox potential of •OH/H_2_O (+ 2.27 eV). According to the relevant reports [46, 47], the VB of SrTiO_3_ is insufficient to oxidize H_2_O into •OH. It indicates that $\bullet {\mathrm{O}}_2^{-}$ and holes are the main oxygen active species for SrTiO_3_/Bi_5_O_7_I nanocomposites in the RhB decolorization, under simulated solar light irradiation. Therefore, the synthesized SrTiO_3_/Bi_5_O_7_I nanocomposite photocatalyst exhibits a much higher photocatalytic performance than that of SrTiO_3_ and Bi_5_O_7_I.

## Conclusions

In summary, novel SrTiO_3_/Bi_5_O_7_I nanocomposites photocatalysts have been designed and fabricated by a solvothermal approach coupled with thermal decomposition. XRD, XPS, and EDS measurements illustrate that the products are indeed SrTiO_3_/Bi_5_O_7_I nanocomposites. UV-vis DRS analysis displays that the SrTiO_3_/Bi_5_O_7_I nanocomposites have a good performance of light absorption. The results of PL spectra show that the recombination of photoinduced electron-hole pairs is obviously inhibited in SrTiO_3_/Bi_5_O_7_I nanocomposites. The obtained nanocomposites show a good stability and a recycling capacity in the photocatalytic process. The as-synthesized SrTiO_3_/Bi_5_O_7_I photocatalysts exhibit a highly efficient photocatalytic property for the degradation of RhB under simulated solar light irradiation, which is superior to that of SrTiO_3_ and Bi_5_O_7_I. The outstanding photocatalytic activity of the photocatalysts is ascribed to the efficient separation and migration of photogenerated charge carriers. The $\bullet {\mathrm{O}}_2^{-}$ and holes are the main oxygen-active species causing the dye degradation. This work could provide insights into the design and development of other excellent photocatalytic materials for environmental and energy applications.

## Abbreviations

BQ: Benzoquinone
CB: Conduction band
DRS: Diffuse reflectance spectra
EDS: Energy-disperse X-ray spectroscopy
EDTA-2Na: Ethylenediaminetetraacetic acid disodium salt
FE-SEM: Field-emission scanning electron microscope
FTIR: Fourier-transform infrared spectroscopy
IPA: Isopropanol
PL: Photoluminescence
RhB: Rhodamine B
UV-vis: Ultraviolet-visible
VB: Valence band
XPS: X-ray photoelectron spectroscopy
XRD: X-ray diffraction
